# Multi‐organ transcriptomic atlas reveals hallmarks of labour

**DOI:** 10.1002/ctm2.70208

**Published:** 2025-02-04

**Authors:** Duan Ni, Ralph Nanan

**Affiliations:** ^1^ Sydney Medical School Nepean The University of Sydney Sydney Australia; ^2^ Charles Perkins Centre The University of Sydney Sydney Australia; ^3^ Nepean Hospital Nepean Blue Mountains Local Health District Sydney Australia

Dear Editor,

1

We present a multi‐organ transcriptomic atlas of labour for unprecedented comprehensive profiling of both organ‐specific and systemic signalling changes associated with labour across maternal and fetal compartments. Labour signifies the concluding phase of pregnancy. While pregnancy is known to induce pronounced maternal and fetal reprogramming,^1^ specific alteration driven by labour remains elusive. In this context, previous studies have predominantly concentrated on individual organ systems,^2^ limited to gene‐level analyses for specific marker gene identification,^3^ and more comprehensive overviews are lacking.

We surveyed Gene Expression Omnibus for all available transcriptomic datasets across both maternal and fetal compartments to collate a multi‐organ transcriptomic atlas, cross‐sectionally comparing labour versus non‐labour (Supporting Information). The atlas contains 16 datasets, spanning six organ systems (maternal blood, subcutaneous fat, visceral fat, placenta, myometrium and cord blood mononuclear cells [CBMCs]), with 392 samples in total (Figure 1).

**FIGURE 1:**
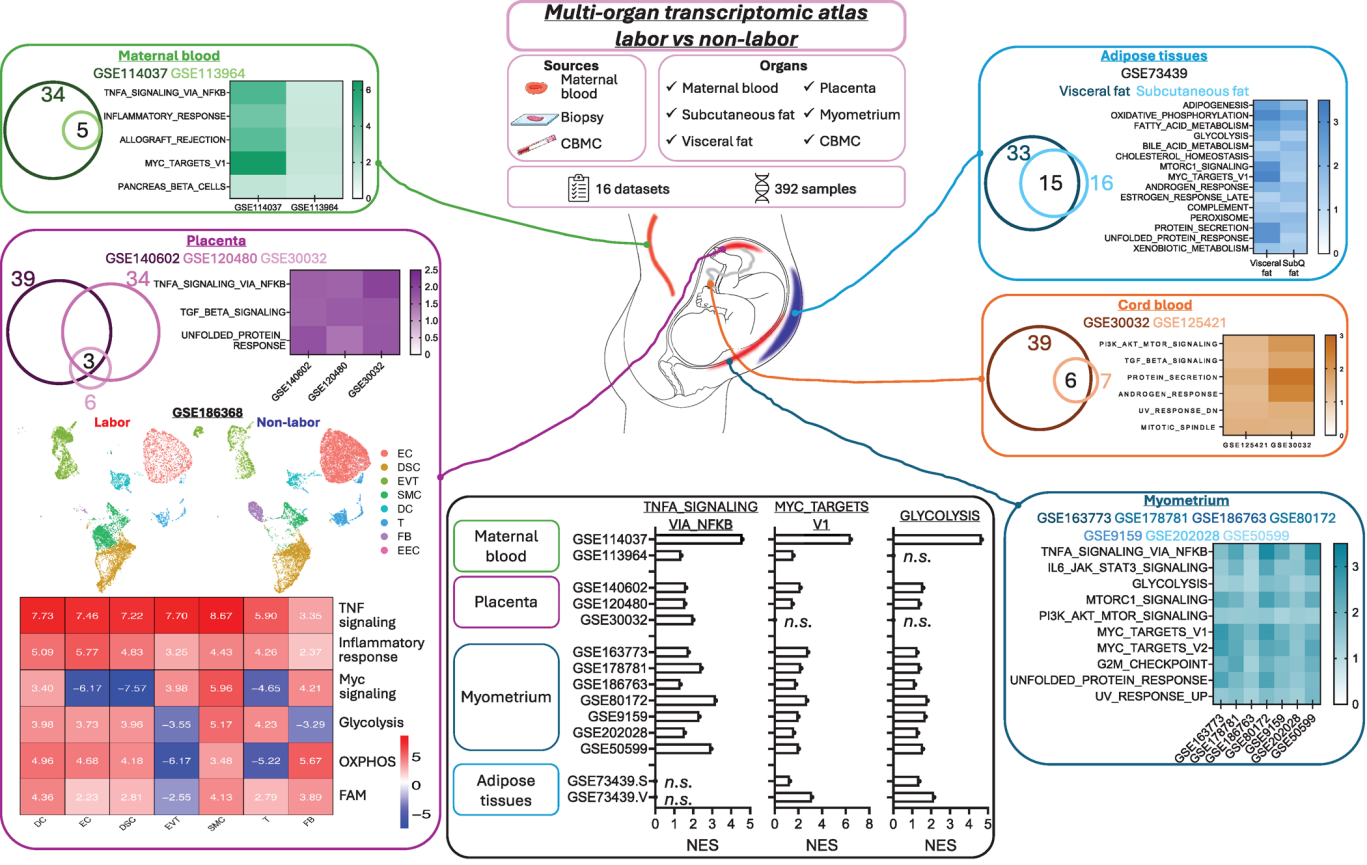
Multi‐organ transcriptomic atlas for labour versus non‐labour. A multi‐organ transcriptomic atlas was curated from 16 independent datasets comprising 392 samples, comparing labour versus non‐labour through six organ systems (maternal blood, green box; maternal subcutaneous fat, maternal visceral fat, cyan box; placenta, purple box; myometrium, blue box; fetal cord blood mononuclear cell [CBMC], brown box). For each organ system, gene set enrichment analysis (GSEA) was run to compare labour versus non‐labour. Pathways consistently enriched in labour conditions were depicted by Venn diagrams and heatmaps, with the colour reflecting the normalized enrichment score for each condition. In the anatomical graphics, organ systems with prominent changes in immune‐related pathways were highlighted in red, the ones with pronounced changes in metabolism‐related pathways were highlighted in blue, and the ones with mild changes were shown in grey. Representative pathways (TNFA_SIGNALING_VIA_NFKB, MYC_TARGETS_V1 and GLYCOLYSIS) changes across organ systems were summarized in the black box.

Extensive analyses like gene set enrichment analysis (GSEA) were run, focusing on pathway‐level changes during labour. For each organ system, we compared the results from different datasets and compiled the most consistent changes. In maternal blood, labour was linked to upregulation of allograft rejection, tumour necrosis factor (TNF)‐NFκB‐related, and Myc‐related signalling (Figure 1). Myc signals were also enhanced in maternal adipose tissues in labour, accompanied by pronounced metabolic changes like enhanced glycolysis, oxidative phosphorylation (OXPHOS) and fatty acid metabolism (FAM) in both visceral and subcutaneous fat (Figure 1).

We next probed the organs directly implicated in labour like myometrium and placenta (Figure 1). Similar to adipose tissues, myometrium exhibited increased glycolysis and Myc signalling. TNF and interleukin (IL)‐6 signalling were higher, possibly induced by mTORC1 activation. These were consistent across seven myometrial datasets.

Labor‐associated immune activation was also found in the placenta, as TNF signalling was consistently higher (Figure 1), aligned with a previous report.^2^ A published single‐cell RNA‐seq (scRNA‐seq) dataset for placental tissues with/without labour was re‐analyzed (Figure 1). As in the original study, eight different cell subsets were identified (endothelial cells, EC; decidual stromal cells, DSC; extravillous trophoblasts, EVT; smooth muscle cells, SMC; dendritic cells, DC; T cells, T; fibroblasts, FB; endometrial cells, EEC). EECs were excluded from downstream analysis due to low cellularity. GSEA found that all cell subsets upregulated the TNF signalling pathway in labour. They also generally displayed more active metabolic profiles, upregulating glycolysis, OXPHOS and FAM. An exception was EVTs, where the aforementioned metabolic signals were downregulated in labour. Furthermore, Myc signalling was increased in labour in DC, EVT, SMC and FB.

Finally, fetal CBMCs were analyzed (Figure 1). Intriguingly, the inflammatory and metabolic pathways were generally not affected in CBMCs. Instead, activation of the PI3K‐Akt‐mTOR pathway and transforming growth factor beta (TGFβ) signalling was found in labour, the latter usually linked to anti‐inflammatory processes.

Overall, our analyses revealed that labour significantly shifted maternal and fetal immunity and metabolism. During labour, activation of pro‐inflammatory TNF signalling was found in all maternal compartments except adipose tissues, which, along with myometrium, showed upregulation in glycolysis. Myc signalling, as a critical nexus within the signalling network, was generally enhanced in labour. Contrarily, fetal CBMCs in labour upregulated the anti‐inflammatory TGFβ signalling.

We next interrogated the effects of parity, known to influence pregnancy.^4^ The aforementioned labour‐associated effects were not confounded (Figure 2A), as a consistent increase in TNF signalling was found for myometrium in primi‐ and multi‐parity pregnancies. Furthermore, in preterm‐impacted and smoking‐affected pregnancies, labour was also linked to TNF pathway activation (Figure 2B).

**FIGURE 2:**
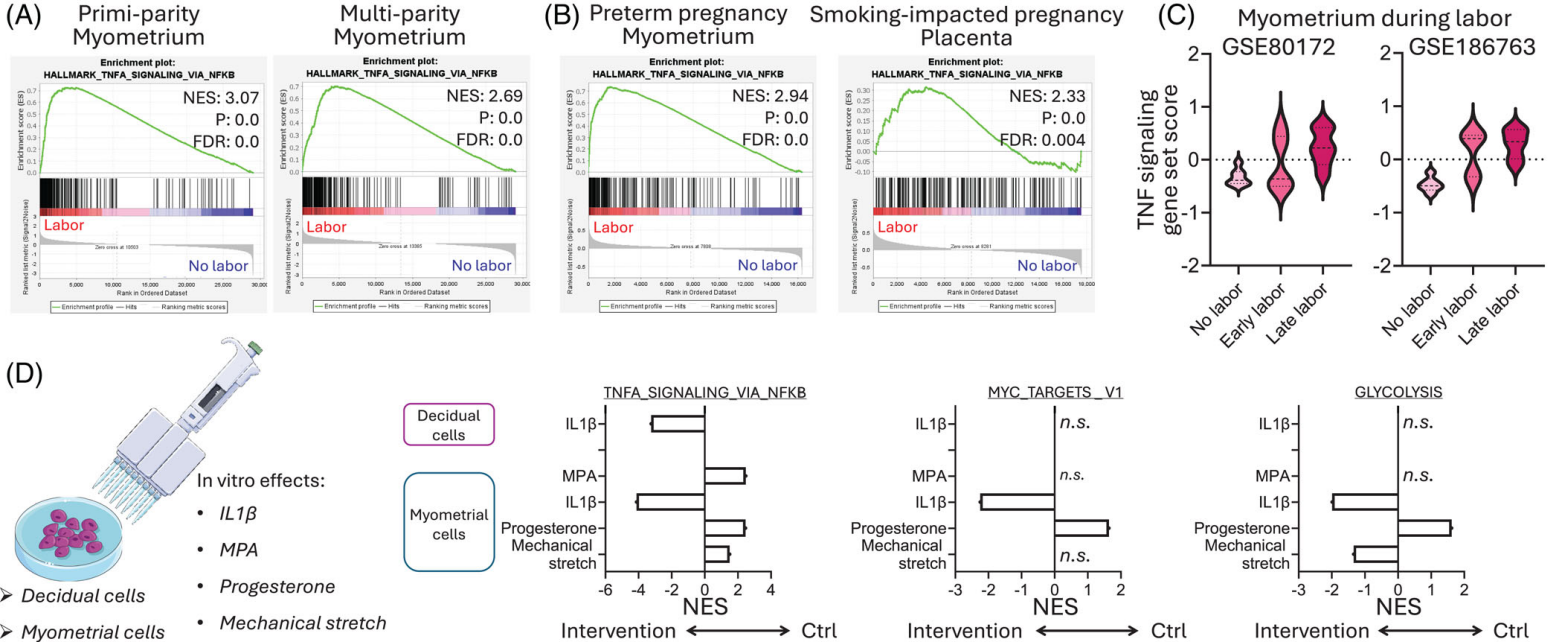
(A) Gene set enrichment analysis (GSEA) showing the enrichment of TNFA_SIGNALING_VIA_NFKB pathway in primi‐ and multi‐parity myometrium. (B) GSEA showing the enrichment of TNFA_SIGNALING_VIA_NFKB pathway in preterm pregnancy myometrium and smoking‐impacted pregnancy placenta. (C) Gene set scores of TNFA_SIGNALING_VIA_NFKB pathway in myometrium throughout the labour time course. (D) Summary of GSEA for representative pathways (TNFA_SIGNALING_VIA_NFKB, MYC_TARGETS_V1 and GLYCOLYSIS) in decidual cells treated with IL‐1β and myometrial cells treated with interleukin (IL)‐1β, medroxyprogesterone acetate (MPA) and progesterone, and undergoing mechanical stretching in vitro.

Longitudinally, gene set variation analysis of two cross‐sectional myometrial datasets from early to late labour unveiled gradual increases in TNF signalling throughout the labour time course (Figure 2C).

Finally, we interrogated specific perturbations associated with labour, including changes in cytokines, hormones and mechanical stress, aiming to decipher the underlying causes for the aforementioned changes. We curated existing datasets analyzing in vitro experiments with decidual or myometrial cells (Figure 2D). Here, IL‐1β expectedly activated the TNF signalling in both decidual and myometrial cells but only promoted the Myc and glycolysis pathways in myometrial cells. Contrarily, exposure to progestin hormones like medroxyprogesterone acetate and progesterone, dampened the TNF signals in myometrial cells. Progesterone also blunted the Myc and glycolysis pathways. In contrast, mechanical stretching of myometrial cells did not increase TNF signalling but promoted glycolysis.

Collectively, these data suggest that lowered progestin signalling in vitro, similar to their “functional withdrawal” during labour,^5^ might lead to the described transcriptomic changes, whilst mechanical stress imposed limited effects.

Here, we present the first multi‐organ transcriptomic atlas comparing labour and non‐labour. We found that labour was linked to increased TNF‐mediated inflammatory signalling in most maternal compartments but associated with enhanced anti‐inflammatory TGFβ signalling in CBMCs. Changes in the maternal compartments seemed to be conserved across various physiological and pathological conditions.

The labour‐associated increase in TNF signalling suggests primarily innate immune activation, excluding adipose tissues. However, both adipose tissues and myometrium exhibited enhanced glycolysis, likely to meet the increased energy demands from labour, like those required for uterine contractions. Increased glycolysis might also drive immune activation,^6^ potentially contributing to the upregulated TNF and Myc signalling in maternal tissues.

Mechanistically, these maternal immunometabolic changes could partly be explained by the fall of progesterone signals during labour, while cellular mechanical stress surprisingly did not play a role. Other perturbations that might shape the described immunometabolic profiles include changes in hormones like oxytocin,^7^ warranting further investigation.

The elevated maternal inflammatory signals coincide with the enhanced maternal leukocytosis upon labour.^8^ Such immune activation might promote maternal immunity against ascending infections during labour and the post‐partum period, a major risk accompanying delivery.

Contrarily, the fetal compartment surprisingly exhibited an anti‐inflammatory phenotype linked to labour. Inflammation in newborns interferes with multiple physiological processes critical for their transition from *in‐utero* to *ex‐utero* life.^9^ The observed anti‐inflammatory TGFβ signalling might thus confer benefits, plausibly linked to the protection against pathologies like respiratory distress syndrome and birth asphyxia^10^ in vaginal delivery.

Finally, transcriptomic atlas‐level analysis, such as ours, inevitably faces limitations, including potential biases from data heterogeneity. While these might be partially adjusted in the original source studies, future research with improved control and validations through complementary modalities like proteomics or functional assays is warranted.

Together, our work provides novel insights towards how labour impacts the mother and the fetus, revealing the hallmarks of this sophisticated process.

## AUTHOR CONTRIBUTIONS

*Concept and design*: Duan Ni and Ralph Nanan. *Acquisition, analysis and interpretation of data*: Duan Ni and Ralph Nanan. *Drafting of the manuscript*: Duan Ni and Ralph Nanan, Critical revision of the manuscript for important intellectual content: All authors. All authors have read and approved the manuscript.

## CONFLICT OF INTEREST STATEMENT

The authors declare no conflict of interest.

## Supporting information



Supporting Information
