# Supplementary material for: Multi‐organ transcriptomic atlas reveals hallmarks of labour
Source: Clin Transl Med. 2025 Feb 4;15(2):e70208. doi: 10.1002/ctm2.70208 (PMC11791753; doi:10.1002/ctm2.70208)
Supplement: Supplementary file 1 — Supporting Information [file CTM2-15-e70208-s001.pdf]

Supplementary information

Materials and Methods

Data curation

We extensively surveyed the Gene Expression Omnibus (GEO) database for studies analyzing labor versus non-labor samples. Notably, studies comparing vaginal delivery (labor) and elective caesarean section (no labor) were also included. Studies without clear case definitions or annotations of labor status were excluded from our analysis. Datasets included in the current study were listed in Supplementary Table S1. For some studies, normalized data are available from GEO, and were downloaded for analysis, while for others, raw data were downloaded and normalized using DESeq2[1] before further analysis. As our comparative analyses were performed within each individual study, different data normalization approaches would not confound the final outcomes. Instead, within each study, the labor and non-labor samples should have been at least in part balanced and accounted for various confounders.

Single cell RNA-seq (scRNA-seq) data for decidual tissue was downloaded from GEO database (GEO ID: GSE186368)[2] and analyzed with *Seurat* (v4)[3].

Data analysis

Gene set enrichment analysis (GSEA) was run with the software (v4.3.2) following the developer's tutorial[4]. GSEA is a computational method to determine whether a given priori defined set of genes exhibit statistical difference between two conditions (i.e. labor vs non-labor here). It takes into account corrections for multiple comparisons as well, as described in[4]. In present study, GSEA were carried out based on the Hallmark Gene Set from GSEA, which summarizes 50 pathways related to major well-defined biological states and processes (e.g. Table S2-4). GSEA was run only within each individual study. After that, results for different studies were compiled and compared together, in an attempt to identify the most consistent changes within/across different organ systems.

Gene set score was calculated using gsva (v1.51.5) package[5] based on the "Human gene set: HALLMARK_TNFA_SIGNALING_VIA_NFKB" gene set from GSEA software. For scRNA-seq analysis, *Seurat* package was used[3]. In brief, data was first quality checked and then integrated with *LIGER*[6] to minimize potential batch effects (alignment = 0.88). Cells were clustered using marker genes reported in the original publication[2]. GSEA was run with the *fgsea* package.

Table S1. Transcriptomic datasets used in the current study (N/A: not available; n.s.: not significant).

**Physiological conditions**						
**Organ system**	**Sample #**	**GEO ID**	**Parity**	**Smoking**	**Ancestral background**	**Ref**
Myometrium	5 labor vs 5 non-labor	GSE163773	Labor: 0±0<br>Non-labor: 0.4±0.24<br>n.s.	N/A	Labor: 2 white, 2 Hispanic & 1 African American<br>Non-labor: 4 white & 1 Asian	7
	8 labor vs 8 non-labor	GSE178781	N/A	N/A	All Asian	8
	6 late labor vs 9 early labor vs 7 non-labor	GSE80172	Non-labor: 0.33±0.20<br>Early labor: 0.57±0.24<br>Late labor: 0.25±0.33<br>n.s.	N/A	N/A	9,10
	3 labor vs 3 non-labor	GSE9159	N/A	N/A	N/A	11
	7 labor vs 5 non-labor	GSE186763	N/A	N/A	N/A	12
	4 labor vs 7 non-labor	GSE202028	Non: 1.14±0.46<br>Labor: 0.75±0.25<br>n.s.	N/A	Labor: 3 Caucasian & 1 Latina<br>Non-labor: 2 Africa American 5 Caucasian	13
	5 labor vs 5 non-labor	GSE50599	N/A	N/A	N/A	14
Subcutaneous fat	15 labor vs 25 non-labor	GSE73439	Matched in original paper	N/A	Balanced in original paper	15
Visceral fat	15 labor vs 25 non-labor	GSE73439	Matched in original paper	N/A	N/A	15
Maternal blood	8 labor vs 8 non-labor	GSE113964	N/A	N/A	N/A	16,17
	8 labor vs 8 non-labor	GSE114037	N/A	N/A	N/A	16,17

Placenta	31 vaginal delivery vs 3 c-section	GSE30032	N/A	(Non-)smoker separated for analysis	N/A	[18]
	6 spontaneous labor vs 6 elective c-section	GSE120480	N/A	N/A	N/A	[19]
	16 labor vs 18 non-labor	GSE140602	N/A	N/A	N/A	-
CBMC	8 vaginal delivery vs 7 c-section	GSE125421	N/A	N/A	N/A	[20]
	22 vaginal delivery vs 2 c-section	GSE30032	N/A	(Non-)smoker separated for analysis	N/A	[18]

Pathological conditions

**Organ system**	**Sample #**	**Disease conditions**	**GEO ID**	**Parity**	**Smoking**	**Ancestral background**	**Ref**
Myometrium	8 labor vs 8 non-labor	Preterm birth	GSE134447	Labor: 1<br>Non-labor: 0.75<br>n.s.	N/A	N/A	[21]
Placenta	16 vaginal delivery vs 7 c-section	Pregnancy exposed to smoking	GSE30032	N/A	Smoking	N/A	[18]

***In vitro* experiments**

**Cell type**	**Sample #**	**GEO ID**	**Ref**
Primary myometrial cells	6 ctrl vs 3 progesterone vs 3 IL-1β	GSE68171	[22]
Primary myometrial cells	9 ctrl vs 9 medroxyprogesterone acetate	GSE22961	[23]
Decidualized human endometrial stromal cells	3 ctrl vs 3 IL-1β	GSE58220	[24]

Table S2. Gene list of the gene set "HALLMARK_TNFA_SIGNALING_VIA_NFKB".

ABCA1	BTG3	CFLAR	DRAM1	FJX1	ICAM1	IL6	KYNU	NFE2L2	PFKFB3	RCAN1	SLC2A3	TNC	VEGFA
AREG	CCL2	CLCF1	DUSP1	FOS	ICOSLG	IL6ST	LAMB3	NFIL3	PHLDA1	REL	SLC2A6	TNF	YRDC
ATF3	CCL20	CSF1	DUSP2	FOSB	ID2	IL7R	LDLR	NFKB1	PHLDA2	RELA	SMAD3	TNFAIP2	ZBTB10
ATP2B1	CCL4	CSF2	DUSP4	FOSL1	IER2	INHBA	LIF	NFKB2	PLAU	RELB	SNN	TNFAIP3	ZC3H12A
B4GALT1	CCL5	CXCL1	DUSP5	FOSL2	IER3	IRF1	LITAF	NFKBIA	PLAUR	RHOB	SOCS3	TNFAIP6	ZFP36
B4GALT5	CCND1	CXCL10	EDN1	FUT4	IER5	IRS2	MAFF	NFKBIE	PLEK	RIPK2	SOD2	TNFAIP8	
BCL2A1	CCNL1	CXCL11	EFNA1	G0S2	IFIH1	JAG1	MAP2K3	NINJ1	PLK2	RNF19B	SPHK1	TNFRSF9	
BCL3	CCRL2	CXCL2	EGR1	GADD45A	IFIT2	JUN	MAP3K8	NR4A1	PMEPA1	SAT1	SPSB1	TNFSF9	
BCL6	CD44	CXCL3	EGR2	GADD45B	IFNGR2	JUNB	MARCKS	NR4A2	PNRC1	SDC4	SQSTM1	TNIP1	
BHLHE40	CD69	CXCL6	EGR3	GCH1	IL12B	KDM6B	MCL1	NR4A3	PLPP3	SERPINB2	STAT5A	TNIP2	
BIRC2	CD80	ACKR3	EHD1	GEM	IL15RA	KLF10	MSC	OLR1	PPP1R15A	SERPINB8	TANK	TRAF1	
BIRC3	CD83	CCN1	EIF1	GFPT2	IL18	KLF2	MXD1	PANX1	PTGER4	SERPINE1	TAP1	TRIB1	
BMP2	CDKN1A	RIGI	ETS2	GPR183	IL1A	KLF4	MYC	PDE4B	PTGS2	SGK1	TGIF1	TRIP10	
BTG1	CEBPB	DENND5A	F2RL1	HBEGF	IL1B	KLF6	NAMPT	PDLIM5	PTPRE	SIK1	TIPARP	TSC22D1	
BTG2	CEBPD	DNAJB4	F3	HES1	IL23A	KLF9	NFAT5	PER1	PTX3	SLC16A6	TLR2	TUBB2A	

Table S3. Gene list of the gene set "HALLMARK_MYC_TARGETS_V1".

ABCE1	CCT7	DEK	ERH	HNRNPA1	KARS1	MYC	PABPC4	PSMA2	RAD23B	RPS6	SNRPD2	TFDP1	XPO1
ACP1	CDC20	DHX15	ETF1	HNRNPA2B1	KPNA2	NAP1L1	PCBP1	PSMA4	RAN	RRM1	SNRPD3	TOMM70	XPOT
AIMP2	CDC45	DUT	EXOSC7	HNRNPA3	KPNB1	NCBP1	PCNA	PSMA6	RANBP1	RRP9	SNRPG	TRA2B	XRCC6
AP3S1	CDK2	EEF1B2	FAM120A	HNRNPC	LDHA	NCBP2	PGK1	PSMA7	RFC4	RSL1D1	SRM	TRIM28	YWHAE
APEX1	CDK4	EIF1AX	FBL	HNRNPD	LSM2	NDUFAB1	PHB1	PSMB2	RNPS1	RUVBL2	SRPK1	TUFM	YWHAQ
BUB3	CLNS1A	EIF2S1	G3BP1	HNRNPR	LSM7	NHP2	PHB2	PSMB3	RPL14	SERBP1	SRSF1	TXNL4A	
C1QBP	CNBP	EIF2S2	GLO1	HNRNPU	MAD2L1	NME1	POLD2	PSMC4	RPL18	SET	SRSF2	TYMS	
CAD	COPS5	EIF3B	RACK1	HPRT1	MCM2	NOLC1	POLE3	PSMC6	RPL22	SF3A1	SRSF3	U2AF1	
CANX	COX5A	EIF3D	GNL3	HSP90AB1	MCM4	NOP16	PPIA	PSMD1	RPL34	SF3B3	SRSF7	UBA2	
CBX3	CSTF2	EIF3J	GOT2	HSPD1	MCM5	NOP56	PPM1G	PSMD14	RPL6	SLC25A3	SSB	UBE2E1	

CCNA2	CTPS1	EIF4A1	GSPT1	HSPE1	MCM6	NPM1	PRDX3	PSMD3	RPLP0	SMARCC1	SSBP1	UBE2L3	
CCT2	CUL1	EIF4E	H2AZ1	IARS1	MCM7	ODC1	PRDX4	PSMD7	RPS10	SNRPA	STARD7	USP1	
CCT3	CYC1	EIF4G2	HDAC2	IFRD1	MRPL23	ORC2	PRPF31	PSMD8	RPS2	SNRPA1	SYNCRIP	VBP1	
CCT4	DDX18	EIF4H	HDDC2	ILF2	MRPL9	PA2G4	PRPS2	PTGES3	RPS3	SNRPB2	TARDBP	VDAC1	
CCT5	DDX21	EPRS1	HDGF	IMPDH2	MRPS18B	PABPC1	PSMA1	PWP1	RPS5	SNRPD1	TCP1	VDAC3	

Table S4. Gene list of the gene set “HALLMARK_GLYCOLYSIS”.

ABCB6	ARPP19	CASP6	COG2	EGLN3	GALK2	GPR87	IGFBP3	ME2	P4HA1	PKP2	RPE	SRD5A3	VCAN
ADORA2B	ARTN	CD44	COL5A1	ELF3	GAPDHS	GUSB	IL13RA1	MED24	P4HA2	PLOD1	RRAGD	STC1	VEGFA
AGL	AURKA	CDK1	COPB2	ENO1	GCLC	GYS1	IRS2	MERTK	PAM	PLOD2	SAP30	STC2	VLDLR
AGRN	B3GALT6	CENPA	CTH	ENO2	GFPT1	GYS2	ISG20	MET	PAXIP1	PMM2	SDC1	STMN1	XYLT2
AK3	B3GAT1	CHPF	CXCR4	ERO1A	GLCE	HAX1	KDELR3	MIF	PC	POLR3K	SDC2	TALDO1	ZNF292
AK4	B3GAT3	CHPF2	CYB5A	EXT1	GLRX	HDLBP	KIF20A	MIOX	PDK3	PPFIA4	SDC3	TFF3	
AKR1A1	B3GNT3	CHST1	DCN	EXT2	GMPPA	HK2	KIF2A	MPI	PFKFB1	PPIA	SDHC	TGFA	
ALDH7A1	B4GALT1	CHST12	DDIT4	FAM162A	GMPPB	HMMR	LCT	MXI1	PFKP	PPP2CB	SLC16A3	TGFBI	
ALDH9A1	B4GALT2	CHST2	DEPDC1	FBP2	GNE	HOMER1	LDHA	NANP	PGAM1	PRPS1	SLC25A10	TKTL1	
ALDOA	B4GALT4	CHST4	DLD	FKBP4	GNPDA1	HS2ST1	LDHC	NASP	PGAM2	PSMC4	SLC25A13	TPBG	
ALDOB	B4GALT7	CHST6	DPYSL4	FUT8	GOT1	HS6ST2	LHPP	NDST3	PGK1	PYGB	SLC35A3	TPI1	
ALG1	BIK	CITED2	DSC2	G6PD	GOT2	HSPA5	LHX9	NDUFV3	PGLS	PYGL	SLC37A4	TPST1	
ANG	BPNT1	CLDN3	ECD	GAL3ST1	GPC1	IDH1	MDH1	NOL3	PGM2	QSOX1	SOD1	GFUS	
ANGPTL4	CACNA1H	CLDN9	EFNA3	GALE	GPC3	IDUA	MDH2	NSDHL	PHKA2	RARS1	SOX9	TXN	
ANKZF1	CAPN5	CLN6	EGFR	GALK1	GPC4	IER3	ME1	NT5E	PKM	RBCK1	SPAG4	UGP2	

References

1 Love, M. I., Huber, W. & Anders, S. Moderated estimation of fold change and dispersion for RNA-seq data with DESeq2. *Genome Biol* **15**, 550, doi:10.1186/s13059-014-0550-8 (2014).

2 Huang, J. *et al.* Deciphering the Intercellular Communication Network of Peripartum Decidua that Orchestrates Delivery. *Front Cell Dev Biol* **9**, 770621, doi:10.3389/fcell.2021.770621 (2021).

3 Hao, Y. *et al.* Dictionary learning for integrative, multimodal and scalable single-cell analysis. *Nat Biotechnol* **42**, 293-304, doi:10.1038/s41587-023-01767-y (2024).

4 Subramanian, A. *et al.* Gene set enrichment analysis: a knowledge-based approach for interpreting genome-wide expression profiles. *Proc Natl Acad Sci U S A* **102**, 15545-15550, doi:10.1073/pnas.0506580102 (2005).

5 Hanzelmann, S., Castelo, R. & Guinney, J. GSVA: gene set variation analysis for microarray and RNA-seq data. *BMC Bioinformatics* **14**, 7, doi:10.1186/1471-2105-14-7 (2013).

6 Welch, J. D. *et al.* Single-Cell Multi-omic Integration Compares and Contrasts Features of Brain Cell Identity. *Cell* **177**, 1873-1887 e1817, doi:10.1016/j.cell.2019.05.006 (2019).

7 Ackerman, W. E. t. *et al.* Molecular signatures of labor and nonlabor myometrium with parsimonious classification from 2 calcium transporter genes. *JCI Insight* **6**, doi:10.1172/jci.insight.148425 (2021).

8 Chen, C., Zhu, S., Bai, L., Sui, M. & Chen, D. The Role of Formyl Peptide Receptor 1 in Uterine Contraction During Parturition. *Front Pharmacol* **12**, 696697, doi:10.3389/fphar.2021.696697 (2021).

9 Stanfield, Z. *et al.* Myometrial Transcriptional Signatures of Human Parturition. *Front Genet* **10**, 185, doi:10.3389/fgene.2019.00185 (2019).

10 Lai, P. F. *et al.* Labour classified by cervical dilatation & fetal membrane rupture demonstrates differential impact on RNA-seq data for human myometrium tissues. *PLoS One* **16**, e0260119, doi:10.1371/journal.pone.0260119 (2021).

11 Weiner, C. P. *et al.* Human effector/initiator gene sets that regulate myometrial contractility during term and preterm labor. *Am J Obstet Gynecol* **202**, 474 e471-420, doi:10.1016/j.ajog.2010.02.034 (2010).

12 Tyagi, S. *et al.* Transcriptomic analysis reveals myometrial topologically associated domains linked to the onset of human term labour. *Mol Hum Reprod* **28**, doi:10.1093/molehr/gaac003 (2022).

13 Dotts, A. J. *et al.* In Vivo Genome-Wide PGR Binding in Pregnant Human Myometrium Identifies Potential Regulators of Labor. *Reprod Sci* **30**, 544-559, doi:10.1007/s43032-022-01002-0 (2023).

14 Chan, Y. W., van den Berg, H. A., Moore, J. D., Quenby, S. & Blanks, A. M. Assessment of myometrial transcriptome changes associated with spontaneous human labour by high-throughput RNA-seq. *Exp Physiol* **99**, 510-524, doi:10.1113/expphysiol.2013.072868 (2014).

15 Mazaki-Tovi, S. *et al.* Characterization of visceral and subcutaneous adipose tissue transcriptome in pregnant women with and without spontaneous labor at term: implication of alternative splicing in the metabolic adaptations of adipose tissue to parturition. *J Perinat Med* **44**, 813-835, doi:10.1515/jpm-2015-0259 (2016).

16 Tarca, A. L. *et al.* Targeted expression profiling by RNA-Seq improves detection of cellular dynamics during pregnancy and identifies a role for T cells in term parturition. *Sci Rep* **9**, 848, doi:10.1038/s41598-018-36649-w (2019).

17 Tarca, A. L. *et al.* Crowdsourcing assessment of maternal blood multi-omics for predicting gestational age and preterm birth. *Cell Rep Med* **2**, 100323, doi:10.1016/j.xcrm.2021.100323 (2021).

18 Votavova, H. *et al.* Deregulation of gene expression induced by environmental tobacco smoke exposure in pregnancy. *Nicotine Tob Res* **14**, 1073-1082, doi:10.1093/ntr/ntr325 (2012).

19 Huusko, J. M. *et al.* Integrative genetic, genomic and transcriptomic analysis of heat shock protein and nuclear hormone receptor gene associations with spontaneous preterm birth. *Sci Rep* **11**, 17115, doi:10.1038/s41598-021-96374-9 (2021).

20 Liu, Y. *et al.* RNA-Sequencing Reveals Gene Expression and Pathway Signatures in Umbilical Cord Blood Affected by Birth Delivery Mode. *Phenomics* **3**, 228-242, doi:10.1007/s43657-022-00086-7 (2023).

21 Fernando, F. *et al.* TBX2, a Novel Regulator of Labour. *Medicina (Kaunas)* **57**, doi:10.3390/medicina57060515 (2021).

22 Lei, K. *et al.* Progesterone and the Repression of Myometrial Inflammation: The Roles of MKP-1 and the AP-1 System. *Mol Endocrinol* **29**, 1454-1467, doi:10.1210/me.2015-1122 (2015).

23 Cordeaux, Y., Tattersall, M., Charnock-Jones, D. S. & Smith, G. C. Effects of medroxyprogesterone acetate on gene expression in myometrial explants from pregnant women. *J Clin Endocrinol Metab* **95**, E437-447, doi:10.1210/jc.2010-1541 (2010).

24 Ibrahim, S. A. *et al.* Inflammatory gene networks in term human decidual cells define a potential signature for cytokine-mediated parturition. *Am J Obstet Gynecol* **214**, 284 e281-284 e247, doi:10.1016/j.ajog.2015.08.075 (2016).
